# Genetic diversity evolution in the Mexican Charolais cattle population

**DOI:** 10.5713/ajas.20.0401

**Published:** 2020-08-30

**Authors:** Ángel Ríos-Utrera, Moisés Montaño-Bermúdez, Vicente Eliezer Vega-Murillo, Guillermo Martínez-Velázquez, Juan José Baeza-Rodríguez, Sergio Iván Román-Ponce

**Affiliations:** 1 Campo Experimental La Posta, Centro de Investigación Regional Golfo-Centro, Instituto Nacional de Investigaciones Forestales, Agrícolas y Pecuarias, Medellín, Veracruz 94277, México; 2 CENIDFyMA, Instituto Nacional de Investigaciones Forestales, Agrícolas y Pecuarias, Colón, Querétaro 76280, México; 3 Facultad de Medicina Veterinaria y Zootecnia, Universidad Veracruzana, Veracruz, Veracruz 91710, México; 4 Campo Experimental Santiago Ixcuintla, Centro de Investigación Regional Pacífico-Centro, Instituto Nacional de Investigaciones Forestales, Agrícolas y Pecuarias, Santiago Ixcuintla, Nayarit 63570, México; 5 Campo Experimental Mocochá, Centro de Investigación Regional Pacífico-Sur, Instituto Nacional de Investigaciones Forestales, Agrícolas y Pecuarias, Mocochá, Yucatán 97454, México; 6 Campo Experimental La Campana, Centro de Investigación Regional Norte-Centro, Instituto Nacional de Investigaciones Forestales, Agrícolas y Pecuarias, Aldama, Chihuahua 32910, México

**Keywords:** Cattle, Effective Number of Ancestors, Effective Number of Founder Genomes, Effective Population Size, Average Complete Generation Equivalent, Generation Interval

## Abstract

**Objective:**

The aim was to characterize the genetic diversity evolution of the registered Mexican Charolais cattle population by pedigree analysis.

**Methods:**

Data consisted of 331,390 pedigree records of animals born from 1934 to 2018. Average complete generation equivalent, generation interval, effective population size (N_e_), and effective numbers of founders (f_e_), ancestors (f_a_), and founder genomes (N_g_) were calculated for seven five-year periods. The inbreeding coefficient was calculated per year of birth, from 1984 to 2018, whereas the gene contribution of the most influential ancestors was calculated for the latter period.

**Results:**

Average complete generation equivalent consistently increased across periods, from 4.76, for the first period (1984 through 1988), to 7.86, for the last period (2014 through 2018). The inbreeding coefficient showed a relative steadiness across the last seventeen years, oscillating from 0.0110 to 0.0145. During the last period, the average generation interval for the father-offspring pathways was nearly 1 yr. longer than that of the mother-offspring pathways. The effective population size increased steadily since 1984 (105.0) and until 2013 (237.1), but showed a minor decline from 2013 to 2018 (233.2). The population displayed an increase in the f_a_ since 1984 and until 2008; however, showed a small decrease during the last decade. The effective number of founder genomes increased from 1984 to 2003, but revealed loss of genetic variability during the last fifteen years (from 136.4 to 127.7). The f_a_:f_e_ ratio suggests that the genetic diversity loss was partially caused by formation of genetic bottlenecks in the pedigree; in addition, the N_g_:f_a_ ratio indicates loss of founder alleles due to genetic drift. The most influential ancestor explained 1.8% of the total genetic variability in the progeny born from 2014 to 2018.

**Conclusion:**

Inbreeding, N_e_, f_a_, and N_g_ are rather beyond critical levels; therefore, the current genetic status of the population is not at risk.

## INTRODUCTION

The development of the Mexican Charolais population began in 1930 in Northern Mexico with several importations of bovines from Charolles, a commune in the Saône-et-Loire department in the region of Bourgogne in Eastern France. The former Charolais breeders association was founded in 1958, whose name later changes to ‘Charolais Charbray Herd Book de México’. The breed’s adaptation ability to different Mexican environments, coupled with good production performance (growth, beef doing ability), contributed greatly to a growing Charolais popularity, making it one of the most predominant beef breeds in the country. It is present in the arid and semiarid regions of the North, the temperate region of the Central Plateau, and the tropical and subtropical regions of the South of Mexico. The current breeders association has a membership of 468 cattlemen, which are distributed in 26 of the 32 states that make up the Mexican territory.

Towards the end of the 1990’s, parameters based on probabilities of gene origin (founder equivalents and founder genome equivalent) to measure genetic variability of wild populations [1,2] were successfully adapted and applied to beef cattle populations [3], on the premise that the classical inbreeding approach to quantify the rate of genetic drift is very sensitive to incomplete pedigree information. Later on, numerous studies were carried out in different countries to monitor the genetic status of horse, donkey, sheep and cattle (dairy and beef) populations to initiate breeding actions if there were possible unfavorable trends. In Mexico, thirty-five beef cattle breeds are officially recognized [4], however, herdbook studies for only two breeds (Simmental and Romosinuano) have been recently conducted [5,6]. Therefore, the aim of this study was to characterize the genetic diversity evolution of the registered Mexican Charolais cattle population by pedigree analysis applying the approaches referred above.

## MATERIALS AND METHODS

### Pedigree data

Genealogical records of cattle born from 1934 to 2018 were provided by the Charolais Charbray Herd Book de México. The data file contained identification numbers of the calf, sire, dam and breeder, and genotype, sex and birth year of the calf.

### Data editing

Initially, progeny with any fraction of Charolais, including Charbray calves, and those with an unknown Charolais proportion were deleted from the data file. Then, the pedigree, consisting of only purebred Charolais animals, was revised to verify that i) sires only appeared as fathers, but not as mothers; ii) dams only appeared as mothers, but not as fathers; iii) calves only appeared as progeny, but not as parent in the same record; iv) no calves were born before neither of their two parents; and v) there were not repeated records. After pedigree verification, the final data file consisted of 331,390 records. Finally, pedigree data were divided into seven periods (or reference populations or subpopulations) of five years each (1984–1988, 1989–1993, 1994–1998, 1999–2003, 2004–2008, 2009–2013, 2014–2018), based on a previous study [7], so as to assess the genetic diversity trend in the last 35 yr. The base year was set to 1984 because a relatively small number of animals were registered before this year; such animals were assumed as unrelated founder individuals.

### Procedures to calculate genetic diversity parameters

Proportion of known ancestors was obtained for the first five generations (parents, grandparents, great-grandparents, and so on) for calves born in the seven time periods. Thus, the second generation for a given animal was assigned a 1.0 if all four grandparents were known, 0.75 if only three were known, and so on [8]. Similarly, generation intervals, defined as the average age of parents when their progeny, upon becoming parents themselves, are born, were calculated for the father-son, father-daughter, mother-son and mother-daughter pathways for progeny born during the seven time periods. The average generation interval was defined as the average of the four pathways. The average inbreeding coefficient was estimated per year of birth, for cattle born from 1984 to 2018. Inbreeding coefficients were calculated using the algorithm described by Sargolzaei et al [9], which is based on an indirect method proposed earlier [10]. Effective population size ($\bar{Ne}$), estimated from individual increase in inbreeding coefficients (Δ*F_i_*), average complete generation equivalent, and effective numbers of founders (*f_e_*), ancestors (*f_a_*), and founder genomes (*N_g_*) were calculated per time period. The $\bar{Ne}$ was estimated as: $\bar{Ne}=\frac{1}{2\bar{\Delta F}}$, where $\bar{\Delta F}$ is the average inbreeding rate of the *n* individuals evaluated [11]. Average complete generation equivalent was calculated for each individual as the sum of (1/2)*^n^* coefficients of all known ancestors, where *n* is the number of generations separating the individual to each known ancestor [12].

The *f_e_* was calculated as: $f_{e}=1/\Sigma_{k=1}^{f}q_{k}^{2}$, where *q_k_* is the probability of gene origin of the *k* ancestor, while the *f_a_* was estimated as: $f_{a}=1/\Sigma_{j=1}^{a}q_{j}^{2}$, where *q_j_* is the marginal contribution of an ancestor *j* that is not explained by other ancestors chosen before. The *N_g_* was calculated as *N_g_* = *1/2f̄*_g_, where *f̄*_g_ is the average coancestry in the reference population. Genetic bottlenecks and random genetic drift were defined as the *f_a_*:*f_e_* and *N_g_*:*f_a_* ratios, respectively. Finally, marginal and cumulated gene contributions of the first fifteen ancestors with the maximum genetic impact were obtained for the latter period (2014 through 2018). The expected marginal contribution of an individual quantifies its contribution to the reference population, which has not previously been explained by greater contributing individuals. A comprehensive explanation of the meaning and computation of parameters based on probabilities of gene origin to evaluate populations based on pedigree information may be found elsewhere [2,3,13].

### Software

Proportion of known ancestors, average complete generation equivalent, average generation interval, effective population size and effective number of ancestors were calculated with the ENDOG software [14]. The software package CFC [15] was used to obtain average coefficient of inbreeding and effective numbers of founders and founder genomes. Gene contribution of ancestors was estimated with the PEDIG program [16].

## RESULTS AND DISCUSSION

### Evolution of the pedigree

The number of animals in the reference population, in the pedigree and with two parents known increased from the first (1984 through 1988) to the fifth period (2004 through 2008), but decreased from the fifth to the last period (2014 through 2018). The number of animals with one known parent rose from the 1984–1988 to the 1994–1998 period, but the opposite was true from the 1994 through 1998 to the 2014 through 2018 period (Table 1).

### Proportion of known ancestors

In general, proportion of known ancestors increased over time periods in generations 1 to 5. In the paternal generation, proportion of known ancestors was very high, oscillating from 0.9919, for the 1984 through 1988 subpopulation, to 0.9999, for the 2014 through 2018 subpopulation. Proportion of known ancestors was also relatively high in the grandparental and great-grandparental generations, ranging from 0.9148 to 0.9965, and from 0.8702 to 0.9898, respectively. In generations 4 and 5, proportion of known ancestors was not as high as in the three most recent generations, as expected, oscillating from 0.7445 to 0.9686, and from 0.5660 to 0.9240, respectively; nevertheless, values across time periods revealed that proportion of known ancestors has substantially improved over time (Table 2). Proportion of known ancestors in generations 1 to 5 of the 2014 through 2018 subpopulation is similar to that reported for three subpopulations of the Brown Cattle of Switzerland (Braunvieh, US-Brown Swiss, and Braunvieh×US-Brown Swiss) [17]. On the contrary, in a more recent study that included American Red Angus, only about 91% of the ancestors were known up to the third generation [18].

### Average complete generation equivalent

Average complete generation equivalent consistently increased across periods, from 4.76, for the first reference population (1984 through 1988), to 7.86, for the last reference population (2014 through 2018). This estimate of pedigree depth, similar to the estimate of pedigree completeness (proportion of known ancestors), indicates that the quality of the pedigree has significantly progressed along the years (Table 2), reaching a high level in the last quinquennium. In a study conducted in France [19], a similar number of complete generation equivalent was observed in the Aubrac (7.8), Charolais (7.9), Limousin (6.7), and Salers (8.0) breed, whereas the Bazadaise (5.8), Blonde d’Aquitaine (5.8), Flamande (5.8), Parthenaise (6.2), Ferrandaise (3.7), Gasconne (3.4), and Rouge des prés bred (4.8) reached a smaller number of complete generation equivalent.

### Inbreeding

**Figure 1:**
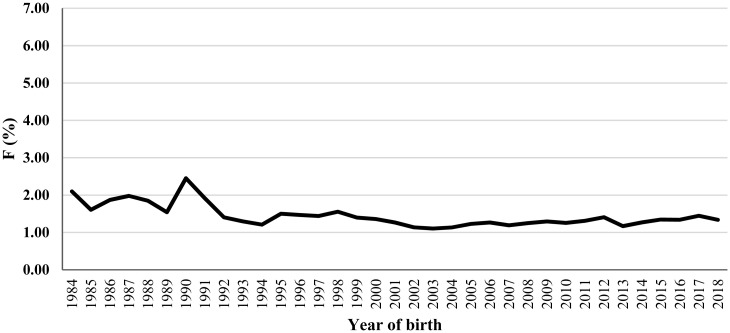
Evolution of inbreeding (F) in the Mexican Charolais cattle population from 1984 to 2018.

During the first seventeen years (1984 through 2000), the inbreeding coefficient showed some fluctuation across the years. For example, inbreeding decreased rapidly from 1984 to 1985, increased moderately from 1985 to 1987, fell once again from 1987 to 1989, rose sharply from 1989 to 1990, and decreased suddenly once again from 1990 to 1992; from 2000 to 2018, however, evolution of the inbreeding coefficient indicates a relative steadiness across time. The slight decrease in inbreeding in the 2000 through 2018 period is attributed to the registration of a higher number of founder animals and outcrossing with imported sires. The variation of the inbreeding coefficient over the first seventeen years could be caused by the relatively small number of the available animals. The average number of animals by year from 1984 to 2000 was 2.3 times smaller than that from 2001 to 2018. However, the coefficient of inbreeding ranged from 0.0110 (2003) to 0.0245 (1990) over the entire interval, showing inbreeding has not been an issue in the registered Mexican Charolais cattle population (Figure 1).

The average inbreeding coefficient of 0.0135 in the latter quinquennium (2014 through 2018) is similar to those reported for the Mexican Simmental population [5], Asturiana de la Montaña [20], the American Limousin population [21], and the Italian Chianina, Marchigiana and Romagnola populations [22]; though, for Japanese Black [23], American Hereford [24] and American Red Angus [18] the average inbreeding coefficient was calculated as 0.05, 0.098 and almost 0.04, respectively.

### Generation interval

The generation interval decreased from the first (1984 through 1988) to the second period (1989 through 1993) for the four parent-offspring pathways, but increased from the third (1994 through 1998) to the seventh period (2014 through 2018). In general, the generation intervals of the two father-offspring pathways were greater than those of the two mother-offspring pathways. In cattle born during the last time period, the average generation interval of the father-offspring pathways was nearly 1 yr. longer than that of the mother-offspring pathways (Table 3). The same pattern has been observed in several dairy (Ayrshire, Guernsey, Holstein, Jersey) [25] and beef cattle populations (Simmental, Charolais, Limousin, Hereford, Angus, Japanese Black) [5,23,26], whose generation intervals of the two pathways from sires were longer than those two from dams, being largely attributed to the use of artificial insemination whereby semen from certain sires were used extensively over time and therefore resulting in sires used beyond their lifespan [25].

### Effective population size

The Mexican Charolais cattle population had a steadily rise in effective size since 1984 and until 2013; but underwent a minor decline from 2013 to 2018. In general, the effective population size was moderately high, fluctuating from 105.0 to 237.1. The effective size of the population in the most recent period was 2.22 times greater than that in the initial period (Table 4). The quality of the pedigree suggests the effective population size was not biased upward (overestimated), mainly that of cattle born in the last period, which had the better pedigree quality (Table 2). Depending on the animal breeding plan, several authors recommend keeping an effective population size between 30 and 250 [27–29]. The effective size of the most recent subpopulation is within this recommended range.

The effective population size maintained by the Mexican Charolais population in the latter period (2014 through 2018) is 2.77 times smaller than that reported for the French Charolais population [19], but is considerably greater than those of many other beef cattle populations, such as the American Hereford [24], the Irish Hereford and Simmental [26], the French Salers and Blonde d’Aquitaine [19], the Japanese Black [23], the Spanish Asturiana de los Valles [13], the Mexican Simmental [5], the Italian Chianina, Marchigiana and Romagnola [22], and the Austrian Braunvieh [30].

### Total and effective number of founders

The population had an increase in the total number of founders from the first (1984 through 1988) to the fourth subpopulation (1999 through 2003), nevertheless, had a decline from the fourth (1999 through 2003) to the seventh subpopulation (2014 through 2018). The effective number of founders decreased across the first three time periods, from 563.7 to 546.2, but exhibited a substantial increment across the last four quinquennia, from 645.2 to 886.8. The relatively great difference between the total and the effective number of founders indicates that some founders were used widely, whereas others contributed little to the population (Table 4). The effective number of founders maintained by the population along the years has been quite big, suggesting that a large proportion of matings among unrelated animals has been possible; therefore, it may explain the milder inbreeding coefficient observed in the present study. The effective number of founders obtained in the current study is greater than that reported by other researchers [5,22,26,30], indicating that the Mexican Charolais cattle population was created from a higher number of individuals in comparison with other beef cattle populations.

### Effective numbers of ancestors and founder genomes

The population displayed a steadily increase in the effective number of ancestors from the first (207) to the fifth period (247); however, exhibited a decrease from the fifth to the sixth period (239); from the sixth to the seventh period the population showed an increase once again, reaching 244 effective ancestors (Table 4). The effective number of founder genomes increased from the 1984 through 1988 (130.1) to the 1999 through 2003 period (143.7), but decreased from the fifth to the seventh period (127.7), revealing some loss of alleles during the last fifteen years (2004 through 2018) (Table 4). The f_a_:f_e_ ratio suggests that the genetic diversity depletion in the last twenty years (1999 through 2018) was partially caused by the formation of genetic bottlenecks in the pedigree. This ratio was 0.381, 0.346, 0.301, and 0.275 for cattle born in the 1999 through 2003, 2004 through 2008, 2009 through 2013, and 2014 through 2018 time periods, respectively. In addition, the N_g_:f_a_ ratio indicates that genetic drift also caused loss of genetic variability in the Mexican Charolais cattle population; this latter ratio was 0.584, 0.552, 0.536, and 0.523, respectively. Even though the Mexican Charolais cattle population lost some genetic diversity, its effective numbers of ancestors and founder genomes are still high compared with the values reported by other authors for Limousin, Abondance and Normande [3]; Swiss brown cattle [17]; Irish Charolais, Limousin, Angus, Hereford, and Simmental [26]; Austrian Simmental, Braunvieh, Pinzgauer, and Grauvieh [30]; and Italian Chianina, Romagnola, and Marchigiana [22].

### Marginal genetic contribution of ancestors

All fifteen Mexican Charolais ancestors with the largest genetic contribution to the genome of the progeny born from 2014 to 2018 were males, with no females having a substantial genetic influence on the population. Among these ancestors, the most influential bull explained 1.8% of the total genetic variability, whereas the two least influential bulls explained 0.8%. The marginal genetic contribution of the first fifteen ancestors was 17.5% of the total genetic variability (Table 5). Contrary to the marginal genetic contribution obtained in this study, McParland et al [26] reported that the most influential ancestor to the Charolais females born in 2004, contributed about 8% of their genes. Similarly, in the Austrian Braunvieh, Pinzgauer, and Grauvieh cattle populations almost 10% of the genes were accounted for by the genetic contribution of only one bull [30]. Overall, the relatively high genetic variability of the Mexican Charolais population may be partially explained by the facts that i) the population derived from a relatively high number of founders, ii) breeders have been importing semen and embryos, and iii) the use of a few bulls has not generally been a practice among breeders.

In conclusion, proportions of known ancestors and aver age complete generation equivalent revealed a highly acceptable quality status (completeness and depth) of the current pedigree. Evolution of the inbreeding coefficient suggests that inbreeding has never been an issue in the population. Interrelationships among f_e_, f_a_, and N_g_ showed that loss of genetic variability was due to genetic bottlenecks in the pedigree and random genetic drift; however, the magnitude of these genetic variability estimates, along with that of the effective population size, indicate that the Mexican Charolais cattle population has a relatively broad genetic base, larger than that of a significant number of dairy and beef cattle populations; therefore, it is not currently in danger of genetic erosion. Expected progeny differences for several traits (e.g., weaning weight, frame score, scrotal circumference, heifer fertility, stayability) have been recently available (last eighteen years) for breeders of the Charolais Charbary Herd Book de México and a widespread use of them is in process. This fact is expected to enhance the use of a few sires with high genetic merit; therefore, future monitoring of the genetic variability of the population and quantification of the effect of inbreeding on economically important traits may be needed.

## Figures and Tables

**Table 1:** Pedigree evolution in the Mexican Charolais cattle population

Item	1984–1988	1989–1993	1994–1998	1999–2003	2004–2008	2009–2013	2014–2018
NAR	7,008	18,706	35,336	49,771	61,990	51,815	48,783
NAP	25,363	52,260	82,929	110,595	132,765	128,122	127,430
NTP	19,221	42,187	70,387	97,013	119,797	115,897	115,756
NOP	281	393	553	408	369	342	326

NAR, number of animals in the reference population; NAP, number of animals in the pedigree; NTP, number of animals with two known parents; NOP, number of animals with one known parent.

**Table 2:** Evolution of proportion of known ancestors and average complete generation equivalent (GE) in the Mexican Charolais cattle population

Period	Generation					GE

	1	2	3	4	5	
1984–1988	0.9919	0.9148	0.8702	0.7445	0.5660	4.76
1989–1993	0.9973	0.9486	0.9083	0.8159	0.6524	5.30
1994–1998	0.9968	0.9404	0.9078	0.8565	0.7144	5.70
1999–2003	0.9997	0.9666	0.9403	0.9066	0.8139	6.31
2004–2008	0.9999	0.9820	0.9618	0.9381	0.8795	6.91
2009–2013	0.9999	0.9925	0.9796	0.9583	0.9162	7.44
2014–2018	0.9999	0.9965	0.9898	0.9686	0.9240	7.86

**Table 3:** Evolution of generation interval (years) by parent-offspring pathway in the Mexican Charolais cattle population

Pathway	1984–1988	1989–1993	1994–1998	1999–2003	2004–2008	2009–2013	2014–2018
Father-son	7.4±0.39	7.0±0.22	6.8±0.15	8.1±0.17	8.2±0.13	8.8±0.16	8.5±0.28
Father-daughter	7.0±0.32	6.0±0.17	6.0±0.13	6.5±0.14	6.6±0.11	6.7±0.13	6.3±0.21
Mother-son	6.5±0.24	5.8±0.11	6.2±0.10	6.3±0.08	6.4±0.08	6.5±0.09	6.6±0.15
Mother-daughter	6.3±0.24	5.5±0.11	5.7±0.09	6.1±0.08	6.2±0.08	6.3±0.09	6.4±0.15
Average	6.7±0.05	5.8±0.03	5.9±0.02	6.4±0.02	6.5±0.02	6.6±0.02	6.5±0.05

**Table 4:** Evolution of genetic variability estimates in the Mexican Charolais cattle population

Estimate	1984–1988	1989–1993	1994–1998	1999–2003	2004–2008	2009–2013	2014–2018
N_e_	105.0±28	157.1±36	164.3±38	226.4±48	232.8±47	237.1±47	233.2±42
F	5,861	9,680	11,989	13,174	12,599	11,883	11,348
f_e_	563.7	562.5	546.2	645.2	712.9	794.0	886.8
f_a_	207.0	216.0	232.0	246.0	247.0	239.0	244.0
N_g_	130.1	133.8	138.3	143.7	136.4	128.0	127.7

N_e_, effective population size based on increase of inbreeding coefficients; f, total number of founders; f_e_, effective number of founders; f_a_, effective number of ancestors; N_g_, effective number of founder genomes.

**Table 5:** The fifteen Mexican Charolais ancestors with the maximum genetic contribution to the calves born from 2014 to 2018

Ancestor	Gender	Year of birth	Number of calves	Genetic contribution (%)	

				Marginal	Cumulated
Ijoufflu	Male	1993	1,034	1.8	1.8
Fandango	Male	1990	146	1.7	3.4
Till	Male	2000	34	1.5	5.0
Tattenhall impeccable	Male	2000	17	1.4	6.4
Flambeau	Male	2000	21	1.3	7.7
Amour de Paris	Male	1965	356	1.3	9.0
Necessaire	Male	1997	1,050	1.1	10.1
Vaillant	Male	1964	343	1.0	11.1
Jumper	Male	1994	833	1.0	12.1
Balmyle Vendetta	Male	2004	559	1.0	13.1
Van Gohg	Male	1984	240	1.0	14.1
Blason	Male	1986	31	0.9	15.0
Quidquid	Male	2000	28	0.9	15.8
Abraham	Male	1975	695	0.8	16.7
Jourdan	Male	1994	767	0.8	17.5
